# P04-10 The Physical activity health paradox - what do we know about physiological mechanisms? (editorial)

**DOI:** 10.1093/eurpub/ckac095.064

**Published:** 2022-08-29

**Authors:** David Hallman, Bart Cillekens, Margo Ketels, Nidhi Gupta, Els Clays, Huysmans Maaike, Holtermann Andreas, Pieter Coenen

**Affiliations:** 1 Department of Occupational and Public Health Sciences, University of Gävle, Gavle, Sweden; 2 Department of Public and Occupational Health, AmsterdamUMC, Amsterdam, The Netherlands; 3 Department of Public Health and Primary Care, Ghent University, Gent, Belgium; 4 National Research Centre for the Working Environment, National Research Centre for the Working Environment, Copenhagen, Denmark

**Keywords:** Occupational physical activity, Editorial, Cardiovascular response

## Abstract

There is strong and consistent evidence that leisure-time physical activity (LTPA) improves cardiovascular health and reduces the risk of all-cause and cardiovascular mortality. Less is known about health effects of occupational physical activity (OPA), and results are not in favor of a beneficial effect on cardiovascular health. Several large-scale prospective studies have found that high occupational physical activity (OPA) is associated with detrimental or no effects on cardiovascular health and mortality. These contrasting associations with cardiovascular morbidity and mortality for LTPA and OPA have coined ‘The Physical activity health paradox'. Although the underlying physiological mechanisms are not established, a theoretical framework was proposed by Holtermann and colleagues (2018). This framework suggests that due to the nature of OPA (i.e. low intensity, long duration, constrained postures, and limited recovery), it may not result in healthy adaptation to the same extent as LTPA, or even lead to unhealthy responses, such as elevated 24-hour heart rate and blood pressure and increased inflammation. Drawing on theoretical models and empirical findings, the aim is to summarize the literature regarding potential physiological mediators of the physical activity health paradox. This also includes a brief summary of our own research based on accelerometer measurements of physical activity with cardiovascular regulation assessed by heart rate and blood pressure in workers with low occupational class and manual work.

